# Altered resting‐state functional connectivity of the anterior cingulate cortex in rats post noise exposure

**DOI:** 10.1111/cns.13896

**Published:** 2022-06-21

**Authors:** Xiao‐Min Xu, Jian Wang, Richard Salvi, Li‐Jie Liu, Yu‐Chen Chen, Gao‐Jun Teng

**Affiliations:** ^1^ Jiangsu Key Laboratory of Molecular Imaging and Functional Imaging, Department of Radiology, Zhongda Hospital Medical School, Southeast University Nanjing China; ^2^ Department of Radiology, Nanjing First Hospital Nanjing Medical University Nanjing China; ^3^ School of Human Communication Disorders Dalhousie University Halifax Canada; ^4^ Center for Hearing and Deafness University at Buffalo Buffalo New York USA; ^5^ Department of Physiology Southeast University Nanjing China

**Keywords:** anterior cingulate cortex, anxiety, depression, functional connectivity, noise‐induced hearing loss

## Abstract

**Aims:**

We aimed to find where and how noise‐induced cochlear hearing loss affects the central nervous system during the early state and identify the neural substrate for aberrant patterns that mediating noise‐related anxiety−/depression‐ like behaviors.

**Methods:**

Broad band noise with 122 dB for 2 hours was conducted to induce hearing loss. We defined 0 day (N0D) and 10 days (N10D) post noise as the acute and sub‐acute period. Behavioral tests (Open field test and light/dark test) and resting‐state fMRI were computed to evaluate emotional conditions and aberrant neural activity. Functional connectivity analysis using the anterior cingulate cortex as a seed was computed to reveal the spatial distribution beyond auditory network during both periods.

**Results:**

Anxiety−/depression‐like behaviors were found in rats with noise exposure. Between‐group analysis revealed that N0D rats displayed widespread reductions in functional connectivity, spanning primary somatosensory cortex, medial geniculate body, inferior colliculus, cingulate cortex, cerebellar lobule comparing with N10D rats and a similar pattern was also occurred in comparison with the control group.

**Conclusion:**

Taken together, an “acoustic‐causing” network accounting for distress and gating of noise exposure related anxiety/depression was proposed.

## Graphical abstract

Emotional behaviors mainly occurred in the acute period of noise exposure. Anterior cingulate cortex‐related neural substrates worked in non‐auditory symptoms. Proposed an” acoustic‐causing” network accounting for distress, pain and anxiety.

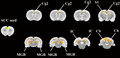

## 1 INTRODUCTION

Noise‐induced hearing loss (NIHL) is known as the second leading cause of sensorineural hearing loss and characterized by the death of outer and inner hair cells, spiral ganglion neurons and the auditory nerve fibers that project to the central auditory system.^1^, ^2^ The World Health Organization estimates that 10% of the world population is exposed to sounds intense enough to cause severe and permanent hearing loss (NIHL).^3^ Military personnel, particularly those in combat, often exposure to excessive noise levels, which were perceived as intolerable loud or even painful.^4^ As the effects of NIHL on the cochlea and peripheral auditory system have been extensively investigated, there is growing evidence that NIHL can induce changes of neural activities outside the classically defined auditory pathway,^5^ which may be related with a specific pattern of neurophysiological and psychological symptoms, such as anxiety and depression.

Anterior cingulate cortex (ACC) has close structural projections to auditory cortex and maybe an ideal brain area for regulating aversive cues (such as annoying noise) and sound‐evoked flight behaviors.^6^ Moreover, it is a relay that interconnects limbic and paralimbic neurons from the frontal cortex, the thalamus, and the amygdala, which contributes to its critical role in cognitive, sensorimotor, affective and visceral functions.^7^ However, the influence of auditory deprivation on ACC remains unclear. In our previous findings and other studies, the role of ACC in hearing loss patients has been proposed.^8^, ^9^ But human studies have often produced diverse and inconsistent findings likely due to subjective ability in performing a given task, the age and gender, gene, lifestyle and environmental circumstances, duration or severity of hearing loss, and ongoing treatments.^10^ We are aware of only one well‐controlled study in which noise‐exposed subjects developed an 8‐dB noise‐induced, temporary threshold shift that recovered within 30 minutes. Sound‐evoked responses in the auditory cortex of the subjects decreased after the narrow‐band noise exposure and the decline was greater in the right than the left auditory cortex.^11^ But it is possible to overcome these confounding variables that plague human studies of NIHL by conducting researches in animals.

In noise exposure animals, traditional electrophysiological technologies were widespread used in the examination of cortical plasticity. In most cases, these measurements were confined to just one subdivision of the central auditory pathways without regarding to how spontaneous activity in one region directly or indirectly affects activity in other auditory or non‐auditory regions.^12^, ^13^, ^14^ Functional MRI is a noninvasive method that can be used to track global brain neurophysiological changes and reveal brain plasticity by the BOLD signals throughout the central nervous system.^15^, ^16^, ^17^ What's more, functional connectivity (FC) analysis, which provides evidence of coincident fluctuations across the whole brain is especially used to reflect the interconnected brain networks.

In this study, we established a rat model with broad intense noise exposure (122 dB), then conduced FC analysis at different time points using the ACC as a region of interest to explore changes of the spatiotemporal correlations among other brain areas, trying to elaborate the potential mechanism of NIHL‐related emotional disturbances.

## 2 MATERIALS AND METHODS

### 2.1 Animal models

A randomized, well‐controlled animal study was performed in Medical School of Southeast University and in accordance with the ethic protocol approved by University Committee for Laboratory Animals. In order to avoid sex differences, forty‐five male Sprague Dawley rats (four weeks of age and weighing between 150–200 g) were used as subjects (Qinglongshan Animal Center, Nanjing, China). Rats were housed in conventional cages with a group of two and maintained under specific pathogen‐free conditions on a 12‐h light/dark cycle (lights in at 7 am) with access to food and water ad libitum. After a one‐week acclimation, the animals underwent noise exposure, auditory brainstem response (ABR) test, behavioral tests and resting‐state functional MRI scanning. We chose 0 day and 10 days post exposure (N0D and N10D) as time points here to observe the early effects of noise exposure and hearing loss.

### 2.2 Intense noise exposure

The rats were placed in separate metal cages (25 cm long × 15 cm wide × 10 cm high) and acclimatized to the setting of noise exposure for 30 min. The rats were awake and unrestrained during the noise exposure. Gaussian noise generated by a System III processor (Tucker‐Davis‐Technologies) was delivered to a power amplifier (YAMAHA, P9500S) and then to a loudspeaker (HG10044XT) located approximately 60 cm above the floor of the animal's cage. The acoustic spectrum of the sound was distributed mainly between 0.1 and 20 kHz as previously reported.^18^ The noise level was adjusted to 122 dB SPL for 2 h and monitored using a 1/4‐inch microphone linked to a sound level meter (Larson Davis 824, Depew, NY, USA). Rats in the Control group underwent the same procedures except that the noise was not turned on.

### 2.3 Bilateral hearing loss measurement

Auditory function was assessed both pre‐exposure and 10 days post noise (N10D) under pentobarbital anesthesia (50 mg/kg, i.p.) by measuring ABR with a TDT system III evoked potential workstation (Alachua, FL, USA), as reported elsewhere.^19^ Responses were recorded with three subdermal needle electrodes that the non‐inverting one was inserted at the vertex in the middle point between the two eyes, and the others attached to the mastoid on each side (ipsilateral as inverting and contralateral as ground electrodes, respectively). During the test, body temperature was maintained at 37.5°C with a thermostatic heating pad. The stimuli were played through a broadband speaker (MF1 from TDT) located 10 cm in front of the animal's head. The electrical activity from the electrodes was amplified 20 times and digitized via a pre‐amplifier (RA16PA) and filtered between 100–3000 Hz. The responses were averaged 1000 times. ABR thresholds were measured from 2 to 32 kHz using tone bursts (1 ms rise/fall time) presented at the rate of 21.1/s. At each frequency, stimulus intensity was performed in a down sequence, starting from 90 dB SPL in 5‐dB steps until the ABR response disappeared. Threshold was determined as the lowest level at which a repeatable wave III response could be obtained.

### 2.4 Open field test (OFT)

We accessed the effect of noise on depression−/anxiety‐like behavior in rats by one of the most popular ethological tests – open field test over a 5‐min period.^20^ The open field apparatus consisted of a square arena (75 cm long × 75 cm wide × 40 cm high) with white walls and a black floor within in a quiet, dimly lit room and the center grid square was lighted (100 lx in the illumination test). Locomotion was measured when the animal was in a prone position, moving the four paws simultaneously. Rearing behavior was tallied when the animal stood upright on its hind legs and lifting its forefeet off the floor.^21^ OFT scoring was conducted by trained and experienced observers who were blind to the experimental treatments. Each rat was tested individually only once after the experimental treatment and the apparatus was cleaned with 70% ethanol before the test of each animal.

### 2.5 Light/dark box (LDB) test

The light–dark box test is a sensitive model to detect activity in disorders related to anxiety−/depression‐ like behaviors, based on the innate aversion of rodents to brightly lit areas and on their spontaneous exploratory behavior in response to a novel environment.^22^ The exploratory activity when an animals exposed to an unfamiliar environment or novel objects showing reflects the combined result of tendencies in new situations. The apparatus consisted of two 34 cm × 24 cm × 24 cm chambers joined together length‐wise with an 8 cm × 8 cm square hole in the middle of the wall separating the two chambers. The white compartment was illuminated with a 100 lx light source, whereas there was no appreciable illumination with the dark box (<2 lx). During the procedures, the rat was first released in the center of the light box (facing away from the opening) and allowed to explore the arena for 5 min. These boxes were equipped with infrared beam sensors and a video camera to record rats' activities. Behaviors in the LDB analyzed included the duration of time spent in the light/dark chamber, latency of first transitions and number of full‐body transitions between chambers. The apparatus was cleaned between sessions with 70% ethanol.

### 2.6 MRI acquisition

Animals were placed in a chamber with 4‐min induction of anesthesia with 5% isoflurane in N_2_/O_2_ (70:30), and maintained throughout scanning with 0.3% iso in N_2_/O_2_ (70:30) plus intramuscular injection of the medetomidine (0.05 mg/kg).^23^, ^24^ Rats were positioned in the MR scanner with a prone position using a bite bar and two ear‐bars, cotton was put in both ears to attenuate scanning noise. Rectal temperature was maintained at 37.5°C with a temperature‐controlled water blanket beneath the rat. The respiratory rate of the rat was monitored continuously during the entire experiment using an MRI‐compatible pulse oximeter and all monitored physiological parameters were within normal ranges and stable. During the scanning, we maintained the breathing rate between 65–75 breaths per min to minimize the influence of anesthesia. Head position was fixed using a home‐built head‐holder with a mouth‐bar and ear‐bars to minimize head motion.

MR examinations were acquired with a preclinal BioSpec 7.0 T animal scanner (Bruker Biospin GembH, Germany) running on a Paravision 5.1 System and a quadrature surface RF coil was used for reception. The BOLD measurements were acquired with a single‐shot gradient‐echo echo planar imaging (GE‐EPI) sequence to acquire multiple slices of images. The parameters were: repetition time (TR) = 2000 ms, echo time (TE) = 18 ms, slices = 21, flip angle (FA) = 90°, for a field of view (FOV) of 3.2 × 3.2 cm, number of averages = 1, matrix = 64 × 64, slice thickness/gap = 1/0 mm, 100 volumes. In addition, a T2‐weighted RARE anatomical images extended anteriorly from the cerebral olfactory bulb to the caudal region of the cerebellum posteriorly ware also collected with the following parameters: TR / TE = 3000/36 ms, slice =21, FOV = 3.2 × 3.2 cm, number of average = 1, matrix = 256 × 256, slice thickness/gap = 1/0 mm, flip angle = 90°.

### 2.7 Data processing

FC was evaluated using seed‐based correlational analysis on a voxel‐by‐voxel basis. Data processing include removing first 10 time points for eliminating the transient blood oxygenation level dependent (BOLD) signal change during the initial acquisition period, slice‐timing correction, realignment, spatial normalization to the standard rat brain atlas and smoothing with a Gaussian kernel of full‐width at half‐maximum of 1 mm. These processing of the fMRI data was carried out with statistical Parametric Mapping software (DPARBI_2.3, http://rfmri.org/dpabi; SPM12, http://www.fil.ion.ucl.ac.uk/spm/). Data were excluded if head movements exceeded 0.1 mm of maximum translation in the x, y, or z directions or 1.0 degrees of maximum rotation about the three axes. An average connectivity among groups were constructed for each animal by placing a voxel‐wise seed in ACC. The resulting FC maps were converted to z scores via Fisher's transform to yield normally distributed data and averaged per animal for use in subsequent analyses. Statistical significance was testing using analysis of variance (ANOVA) among the CN, N0D, N10D (*n* = 15 per group). A *p* < 0.05 with a minimum cluster size of 10 voxels was regarded as significant. What's more, there was an a priori interest in all between‐group comparisons (CN vs. N0D, CN vs. N10D, and N0D vs. N10D). A correction for multiple comparisons was performed, resulting in a corrected threshold of *p* < 0.05/3. Both motion parameters resulting from the realignment and the global signal time course were regressed out during this analysis to improve the specificity of the FC.

### 2.8 Statistical analysis

GraphPad Prism 9.0 software (GraphPad Prism Inc., San Diego, CA, USA) was used for statistical analysis. We used Shapiro–Wilk to do tests for normality, and found some data did not show normal distribution. Thus, a non‐parametric equivalent with Kruskal‐Wallis test was conducted in subsequent statistical analysis.

## 3 RESULTS

### 3.1 ABR thresholds and confirmation of hearing loss

**FIGURE 1:**
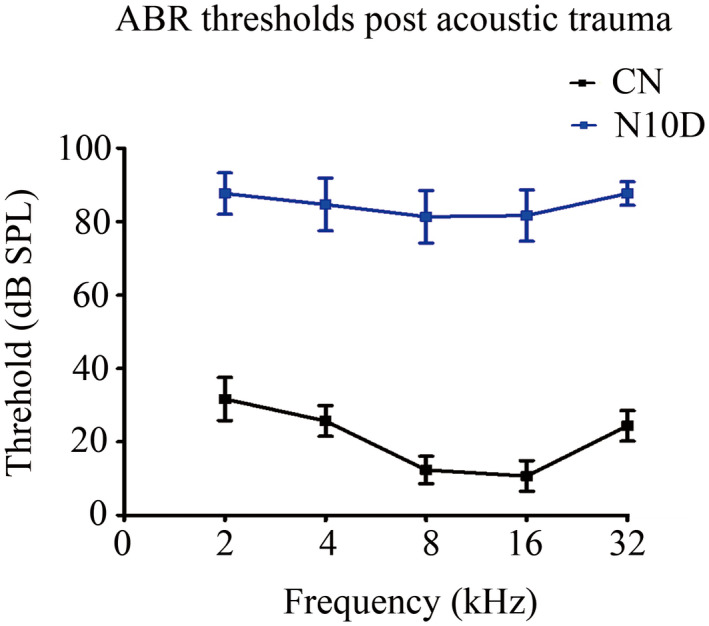
Noise exposure induced ABR threshold shift. Mean ABR thresholds of different frequency 14 days post intense noise were shown, including 2, 4, 8, 16, 32 kHz. Bars show the arithmetic mean and vertical lines represent the standard deviation

Each rat has normal hearing ability during the baseline time, and the averaged threshold was found to be 85.42 ± 6.46 dB SPL in N10D rats, which was obviously higher than the value of 20.08 ± 5.85 dB SPL for the CN group (mean ± SD, *n* = 30, *p* < 0.001). Figure 1 shows ABR thresholds of N10D rats were 60–70 dB higher than the CN group at all frequencies post exposure.

### 3.2 Open field behavior

**FIGURE 2:**
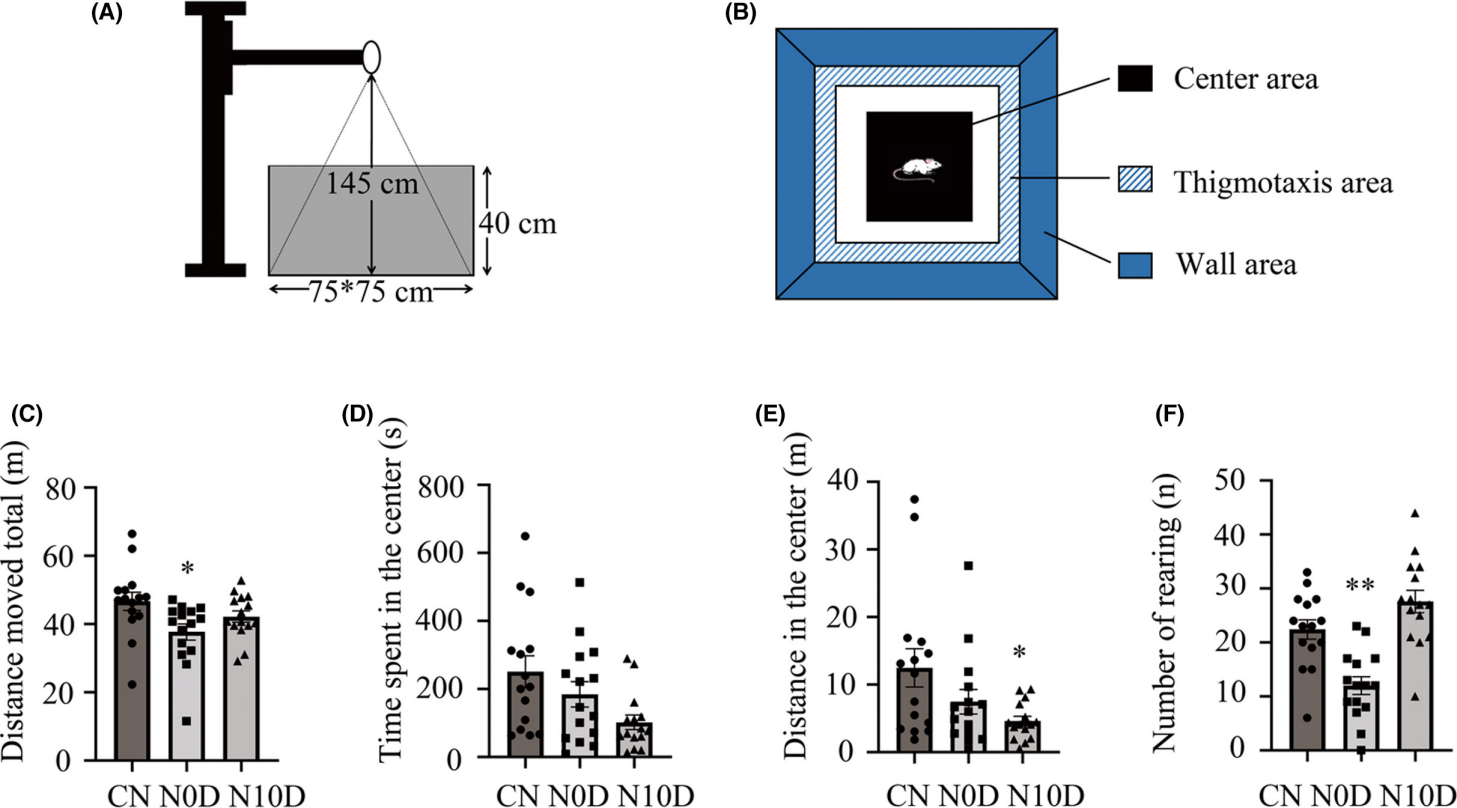
Behaviors in the open field arena during acute and sub‐acute periods. (A) Illustration of the open field test apparatus. (B) Upper view of open field and assessment of the behaviors in the open field test. 35 cm × 35 cm square at the center of the arena (75 cm × 75 cm) was defined as the center area. Each group of rat was placed in the open field apparatus and their distance moved total (C), time spent in the center area (D), distance traveled in the center (E) and number of rearing (F) were measured. *; *p* < 0.05; **p < 0.01. Bars show the arithmetic mean and vertical lines represent the standard error of the mean

During the early stage, we defined N0D as the acute period and N10D as the sub‐acute period. In the acute period, N0D rats were less active than CN rats, displaying a lower distance traveled (*p* = 0.010, Figure 2C). No significance among three groups was observed in terms of time spent in the center (Figure 2D). N10D rats also moved less distance in the center of the apparatus than CN rats (*p* = 0.023, Figure 2E). Number of rearing among three groups was at significant p value and the N0D rats exhibited fewer rearing than CN ((*p* = 0.007) and N 10D group (*p* < 0.001, Figure 2F).

### 3.3 Light/dark box behavior

**FIGURE 3:**
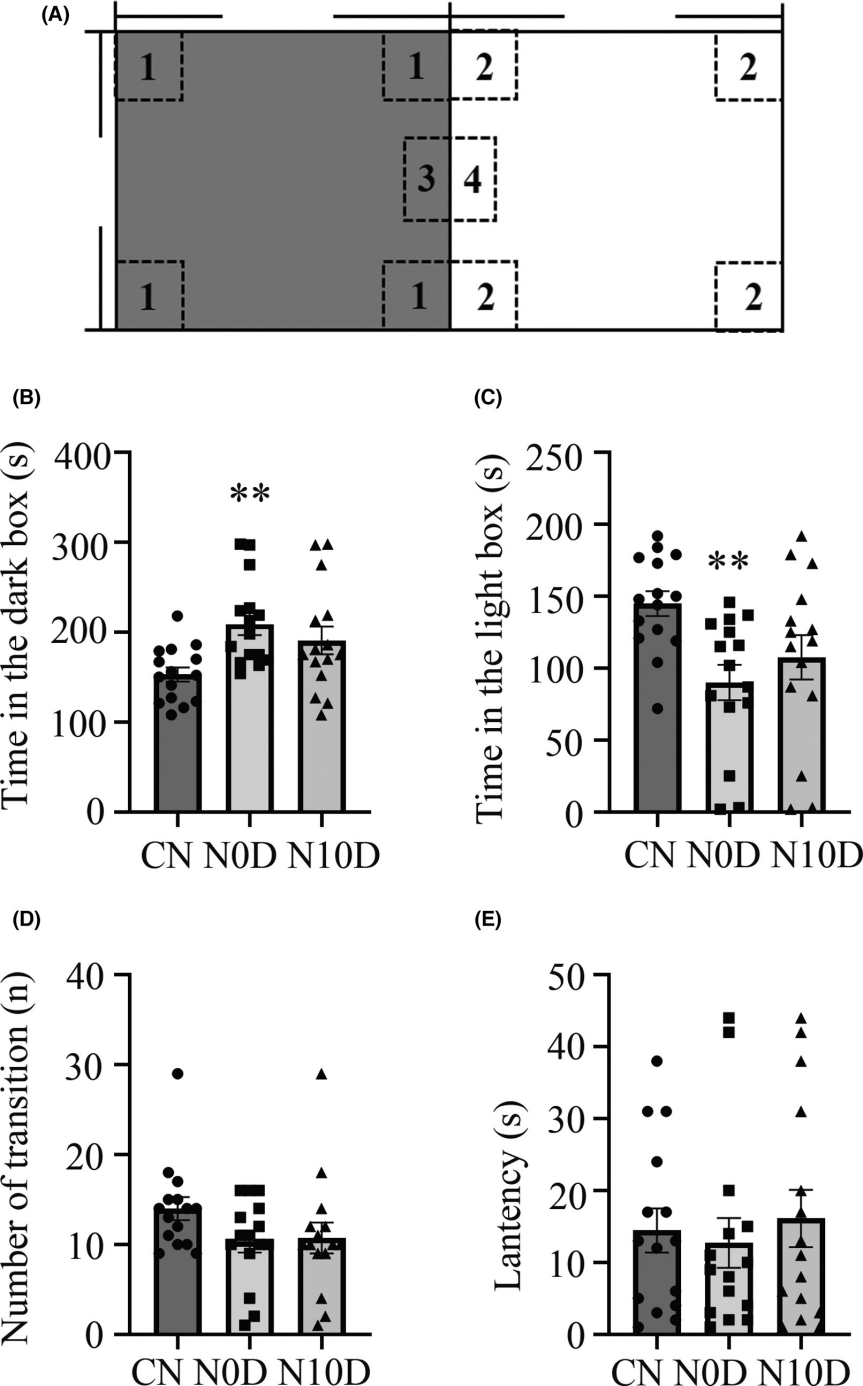
The effects of noise exposure on behaviors in the light/dark box. (A) Schema of the light and dark box (area of dark compartment is shaded). Rectangle box (34 × 24 cm) used with infrared sensors: Zone 1/2 ‐ corners in the dark/light compartment; Zone 3/4 ‐ door zone in the dark/light. (B) Time spent in the dark box. (C) Time spent in light compartment. (D) Number of the transition. (E) Latency of first transition to the other area. **; *p* < 0.01. Bars show the arithmetic mean and vertical lines represent the standard error of the mean

Behaviors in the LDB test was assessed in three groups of animals and the results are presented in Figure 3. An acoustic exposure effect was observed across different time points. The CN rats spent a significantly shorter time in the dark box than N0D (*p* = 0.006) group and showed a tendency in N10D (*p* = 0.142) rats (Figure 3B). However, no significant effects of noise exposure for number of transition and latency to enter the dark box were observed among groups (Figure 3D,E). Overall, these data were in accordance with OFT, suggesting rats were much more anxious/depression in the acute period and sustained little till 10 days later.

### 3.4 Functional connectivity analysis

**FIGURE 4:**
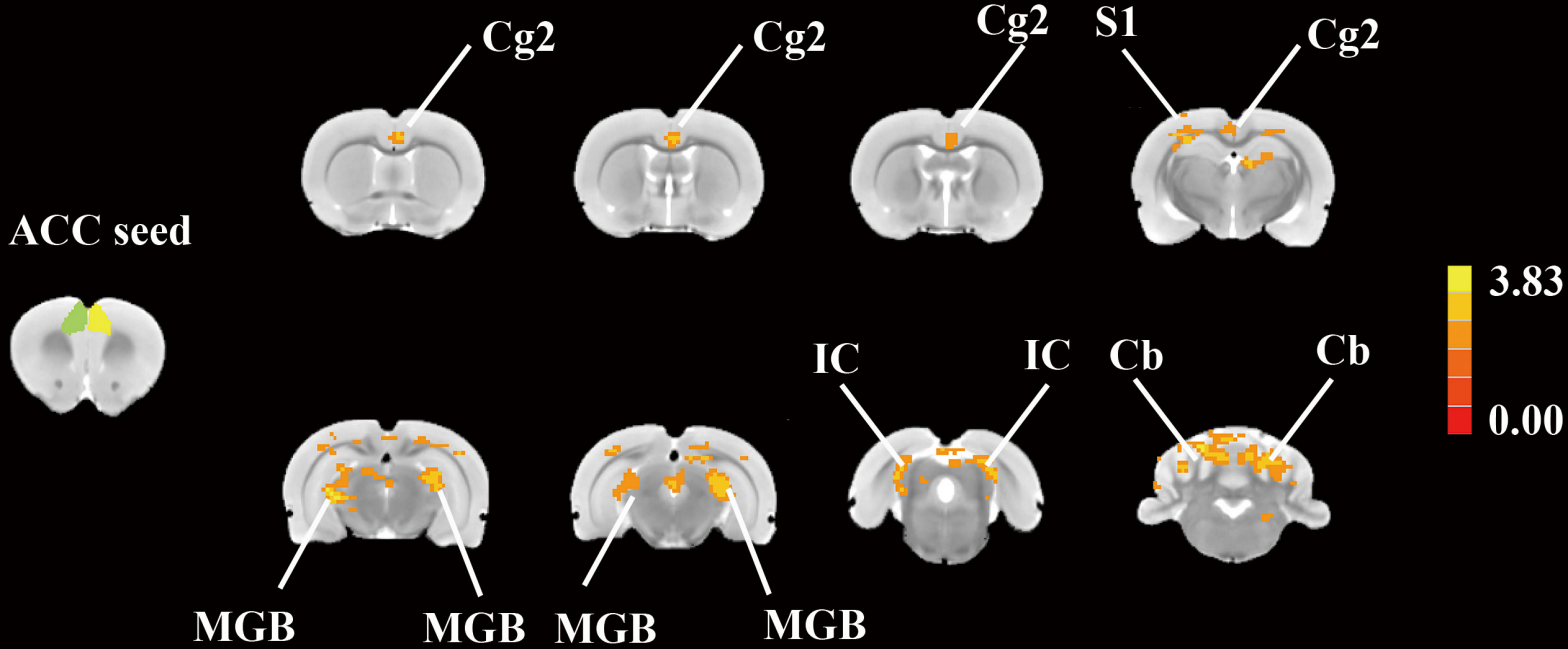
One‐way ANOVA of ACC resting‐state connectivity analysis among three groups (A *p* < 0.05 with a minimum cluster size of 10 voxels, Alphasim corrected.). Five clusters were revealed significant main effect: Cg2, cingulate cortex, area 2; S1, primary somatosensory cortex; MGB, medial geniculate body; IC, inferior colliculus; Cb, cerebellar lobule. Scale bar shown in the right; corrected t‐values ranged from 0.00 to 3.83

**FIGURE 5:**
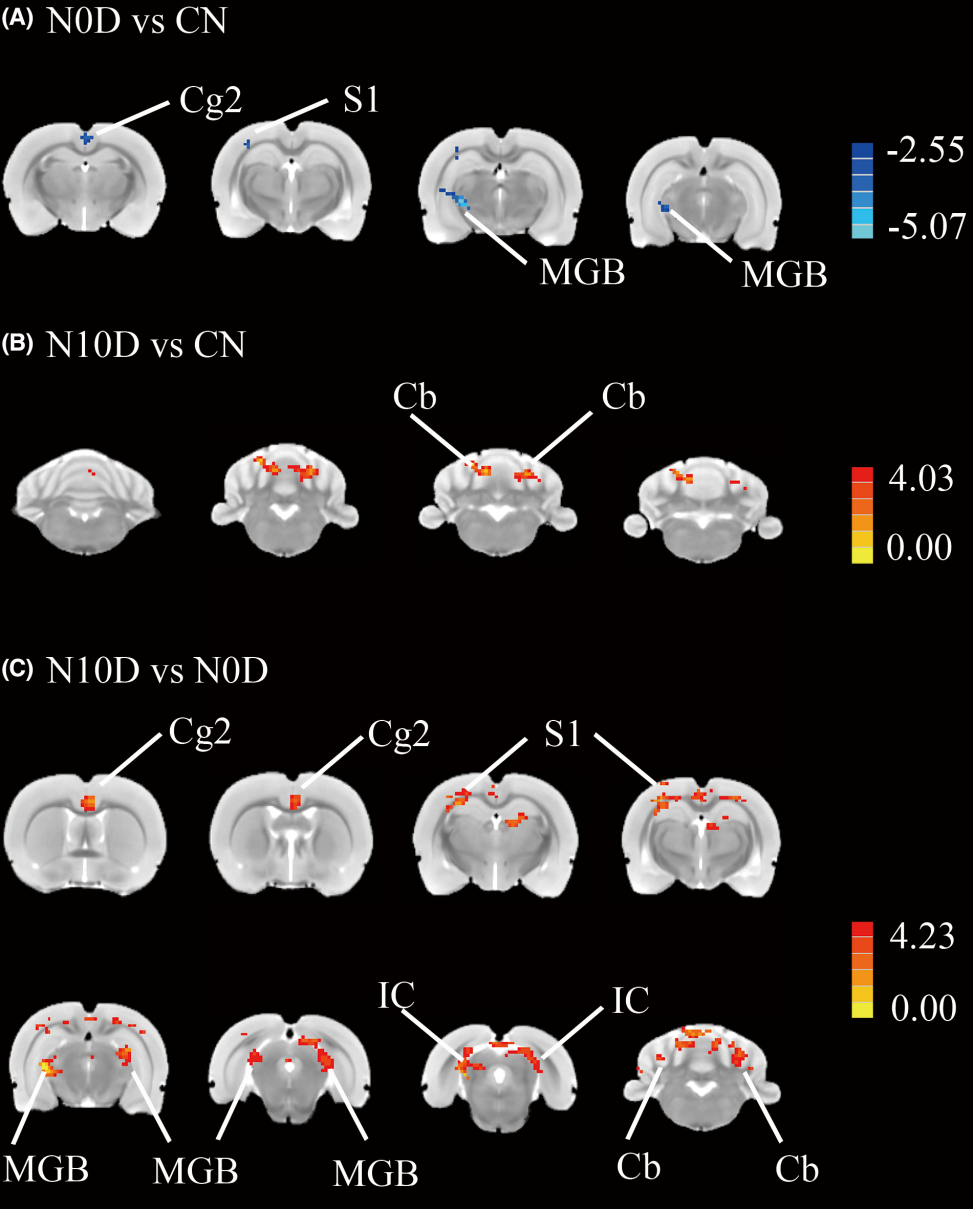
Between‐group contrast maps in FC with the ACC (*p* < 0.05/3, corrected for multiple comparisons). FC maps of cold color shows brain regions where acoustic exposure depressed the functional connectivity with ROI placed in ACC while heat color represents regions of increased functional connectivity. (A) N0D vs CN; (B) N10D vs CN; (C) N10D vs N0D. (Cg2, cingulate cortex, area 2; S1, primary somatosensory cortex; MGB, medial geniculate body; IC, inferior colliculus and Cb, cerebellar lobule.) Scale bar shown in right, showing the corrected *t*‐values

Representative structural images were shown in Figure S1, no obvious hemorrhage exist. To determine if noise altered FC, an unbiased, whole brain survey to test whether rs‐cortical network interaction with the ACC was computed to identify regions where significant difference occurred in the FC maps for noise and control conditions. As shown in Figure 4, mean FC maps were compared across CN, N0D and N10D groups using a one‐way ANOVA (*p* < 0.05, GFR corrected). Subsequent between‐group contrasts confirmed that noise‐related changes in FC of ACC were predominantly restricted to anxiety rats. Notably, N0D animals showed significant and widespread reductions in FC compared with N10D rats, spanning primary somatosensory cortex (S1), medial geniculate body (MGB), inferior colliculus, cingulate cortex, area 2 (Cg 2), cerebellar lobule (Cb) (Figure 5C, Table 1, *p* < 0.05/3 corrected for multiple comparisons). A qualitatively similar pattern of weaker connectivity was also observed when comparing N0D and CN rats, although in this case differenced were somewhat less spatially extensive (Figure 5A, Table 1, *p* < 0.05/3 corrected for multiple comparisons) and increases FC was observed between ACC and Cb in the sub‐acute period (Figure 5B, Table 1, *p* < 0.05/3, corrected for multiple comparisons), suggesting a “depression‐enhancement” rule for noise exposure.

**TABLE 1:** Significant brain regions of functional connectivity analysis at two time points using the anterior cingulate cortex as the seed among groups

Brain region	Coordinate	Cluster size	*t* value
N0D vs CN			
MGB	(39, 6, −45)	57	−5.0725
S1	(45, 48, −36)	23	−3.756
Cg2	(0,54, −24)	21	−3.53
N10D vs CN			
Cb	(27, 45, −105)	85	3.7184
Cb	(−30, 24, −129)	23	3.2625
N10D vs N0D			
MGB、IC	(48, 15, −51)	526	4.2296
S1	(48, 48, −39)	481	3.8631
MGB	(−12, 27, −30)	119	3.2389
Cg2	(0, 42, 0)	64	3.6025

*Note*: The threshold was set at *p* < 0.05/3, corrected for multiple comparisons. Cg2, cingulate cortex, area 2; S1, primary somatosensory cortex; MGB, medial geniculate body; IC, inferior colliculus and Cb, cerebellar lobule. N0D, 0 day post noise; N10D, 10 days post noise; CN, control.

## 4 DISCUSSION

Our results indicated that: (1) A rat model of hearing loss induced by intense noise showed anxious−/depression‐like behaviors (e.g., freezing still and much more prefer to dark environment) in acute and sub‐acute periods; (2) Comparing to the CN group, NIHL exhibited a distinct network signature of widely distributed changes in temporally coherent FC with the seed of bilateral ACC; (3) Unlike the central gain of tinnitus, NIHL depressed the whole brain functional connectivity immediately and increased the FC in sub‐acute time, which may reflected the potential plasticity process.

Noise exposure can be annoying and stressful, moreover, it is also known to affect mood, cognitive performance, and attention.^25^ It can trigger the release of glucocorticoids by activation of the hypothalamic–pituitary–adrenal axis,^26^ which is involved in oxidative stress mechanism. What's more, the effects of oxidative stress induced by noise is transient and disappeared after 7 days, as indicated by the changes of stress hormones, such as serum corticosterone level.^27^ Nonetheless, increased reactive oxygen species generation in cochlear fluids and tissues was well documented and much higher 1 h post noise and persisted for 7–10 days or longer after the cessation of the noise, as oxidative stress overwhelmed the beneficial potential of autophagy in outer hair cells and led to physiological function deficits,^28^, ^29^ and the most distinctive difference is the noise intensity. Our experiments confirmed anxiety−/depression‐like behaviors in N0D rats, including less and slower ambulation, lower number of rearing and more time in the dark environment, suggesting the stress factor (intense noise) might induce these avoidance/numbing behaviors at the acute period. While only a decrease of the distance and time in the center was observed in N10D rats in OFT, as well as no significance in LDB test, indicating weakened effects of noise exposure and hearing loss may start to work.

Functional connections, which are found both humans and animal's measures correlations in the BOLD time‐series of activations in different regions to assess the neuronal oscillations that occur synchronously over spatially disrupted networks. Human imaging studies have identified many inconsistent and contradictory brain sites, but it remains to be clear what exactly causes these conditions. Although previous researches indicated that increased excitatory neuronal transmission and disabled inhibitory synaptic transmission met the metabolic demands, which contribute to vasodilation post noise,^30^, ^31^ others also reported a reduction in cerebral vascular reactivity because of the disputed structural integrity of the microvasculature,^32^ neural loss and abnormal cell signaling^33^ in the acute period, while these will alter during sub‐acute time because of recovery, which is consistent with our FC data of “depression‐enhancement” tendency.

Besides, the ACC, which is involved in the appraisal and expression of conditioned response often coactive during emotional processing and is reported to play an essential role in the pathophysiology of anxiety/depression.^34^ A longitudinal research demonstrated that functional connections of ACC can predict the treatment response for depression.^35^ The theory of “inefficient high‐order control” is popular among human imaging studies that task‐related activation of ACC was greater for higher level of anxiety but with weaker functional connectivity between ACC and lateral prefrontal cortex.^36^, ^37^ Furthermore, our findings showed decreased ACC‐Cg2 functional couplings during the acute time, which is consistent with above theory, indicating abnormal integration or “disconnection” between these two brain regions.^38^ Magnetic resonance spectroscopy discovered a dysregulated balance of excitatory/inhibitory cortical activities and increase in neuronal excitation in the cortex may be related to heightened anxiety.^39^ In addition, intracellular recording study found reduced activity of cortical high‐frequency electroencephalogram components, which were relevant to gamma oscillations, which was framed by synchronized spiking of inhibitory interneurons in hereditary deafness.^40^ Given that (1) anxiety/depression rating is positively correlate with neural activities in sensory processing regions^41^; (2) reduced gray matter density in bilateral primary somatosensory cortex is found in anxiety patients^42^; (3) there is a significant decrease parvalbumin‐containing gamma‐aminobutyric acid‐ergic interneurons in somatosensory cortex in deaf mice,^40^ it is reasonable to assume that the decreased FC between ACC and S1 is convincing. As reported in previous structural MRI,^43^ the decreased ACC‐thalamus (including MGB) coupling in the current study may be a result of the structural abnormality of thalamus. The IC‐centrally located in the auditory pathway‐is an obligatory relay station for all information ascending from brainstem nuclei to thalamus and cortex, and was always known as a region encodes sound frequency,^44^ the involvement in the acoustic‐causing network is under our expectation. Whether changes in activity are compensatory or a driver of impairment is less clear.

Interestingly, the cerebellum, a traditional motor structure, is involved in this disrupted network, which is in a general agreement with long‐term metabolic changes in cerebellum following acoustic exposure using a gas chromatography mass spectrometry‐based metabolomics platform,^45^ suggesting a more widespread effect of intense noise in disturbing the balance between excitatory and inhibitory amino acids during sub‐acute period. Consistent with this view, N10D showed increased ACC‐Cb coupling compared to CN and N0D rats. Moreover, meta‐analysis^46^ and cerebellar‐dependent associative learning task^47^ implies the cerebellum's important role in behaviorally inhibition and anxiety vulnerability. And patients with lobule VIII strokes underwent unpleasant feelings.^48^ Combining all of these, the cerebellum acts as a gain control mechanism and its new role in anxiety/depression has achieved acceptance.

Though intriguing, our fMRI data does have some limitations. Firstly, functional connectivity can change due to internal and external stimulations,^49^, ^50^ so that the impact of task performance on the similar brain regions is needed to further delineation. Secondly, the seed‐based analysis investigate limited number of interactions, hence whole brain connectivity analysis, such as graph theory‐based network analysis, may figure out the key‐region alterations in FC.^51^ Thirdly, since the FC‐MRI is an indirect and relative measures of neural activity fluctuations,^52^ it is necessary to combine new techniques, such as optical fiber‐based recordings, to understand the underlying mechanism of acoustic trauma induced psychological complications from multiple aspects.

## 5 CONCLUSION

Our finding is a powerful imaging evidence of intense noise‐induced anxiety/depression at different periods, proposing an “acoustic‐causing” network and leading to a deeper understanding of potential neural circuit. We aimed to provide treatment biomarkers and key therapeutic time window for emotional disorders in the future.

## CONFLICT OF INTEREST

The authors declare that there is no potential conflict of interest regarding the publication of this paper.

## Supporting information


**Figure S1** Representative T2‐weighted images for (A) CN group, (B) N0D group, (C) N10D group. (N0D, 0 day post noise; N10D, 10 days post noise)

## Data Availability

The data that support the findings of this study are available from the corresponding author upon reasonable request.
