# Supplementary material for: Altered resting‐state functional connectivity of the anterior cingulate cortex in rats post noise exposure
Source: CNS Neurosci Ther. 2022 Jun 21;28(10):1547–56. doi: 10.1111/cns.13896 (PMC9437238; doi:10.1111/cns.13896)
Supplement: Supplementary file 1 — Figure S1 Representative T2‐weighted images for (A) CN group, (B) N0D group, (C) N10D group. (N0D, 0 day post noise; N10D, 10 days post noise) [file CNS-28-1547-s001.docx]

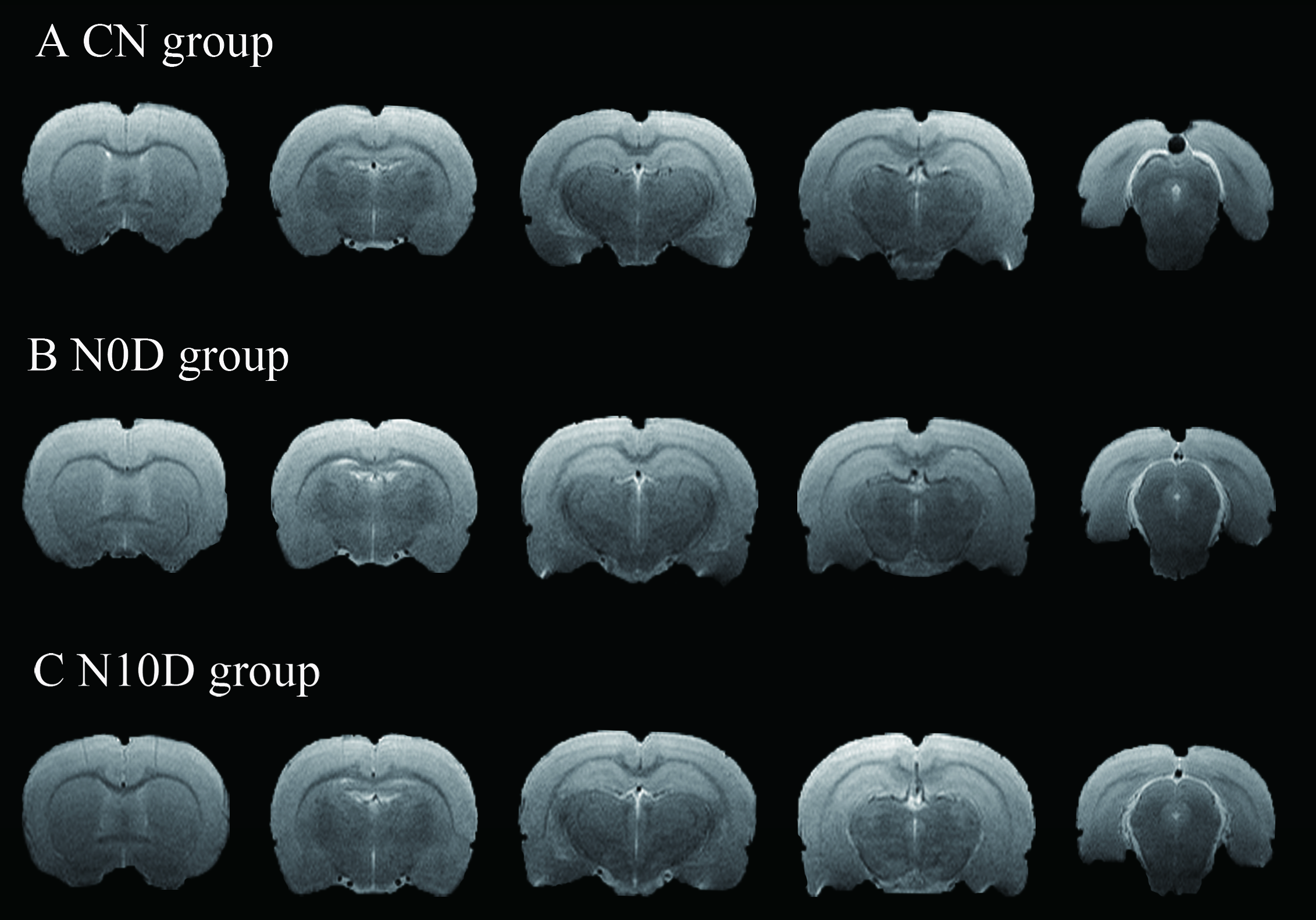


**Figure S1.** Representative T2-weighted images for (A) CN group, (B) N0D group, (C) N10D group. (N0D, 0 day post noise; N10D, 10 days post noise)
